# Data on changes in renal calyceal pattern among renal donors using CT angiography

**DOI:** 10.6026/97320630017699

**Published:** 2021-07-31

**Authors:** M Kumaresan, A Sangeetha, PK Sankaran, Gunapriya Raghunath, Balaji Karunakaran, Yuvaraj Maria Francis, T Siva, J Vijayakumar, Madhan Krishnan

**Affiliations:** 1 Department of Anatomy, Saveetha Medical College, Saveetha institute of medical and technical sciences, Thandalam, Chennai - 602 105, India; 2 Department of Physiology, Saveetha Medical College, Saveetha institute of medical and technical sciences, Thandalam, Chennai - 602 105, India; 3 Department of Anatomy, All India Institute of Medical Sciences, Mangalagiri - 52008, Andhra Pradesh, India; 4 Department of Anatomy, Sri Ramachandra Institute of Medical Sciences, Porur, Chennai, India; 5 Department of Biochemistry, Saveetha Dental College and Hospitals, Saveetha Institute of Medical and Technical Sciences, Chennai, Tamil Nadu, India

**Keywords:** Renal, calyceal pattern, CT angiography, urology

## Abstract

It is known that the recovery period after laparoscopic surgery is quicker than open surgery. Therefore, it is important to know the variations of the renal calyceal pattern prior to the laparoscopic surgery during renal surgeries. We analyzed the calyceal system in kidney donors using 64-Slice Computed Tomography Angiography. A total 99 healthy kidney donors were included to study the pattern of pelvicalyceal pattern and to classify further into bi-calyceal, tri-calyceal and multi-calyceal. This study found that bi-calyceal pattern is the most common pattern and further this pattern was more in right side in males and left side in females. The numbers of minor calyces were significantly more in right multi-calyceal pattern than left multi-calyceal, tri-calyceal and bi-calyceal patterns. The association of occurrence in these patterns was significant in males with strong association and insignificant in females. A detailed description of intrarenal arterial patternand its relationship with calyceal pattern could give great significance in renal transplantation and also for other urological procedures.

## Background:

The kidneys on coronal section have an external cortex and an internal medulla. The columns of Bertini separate the medulla into pyramids. The apical portion of the pyramids extending into the minor calyces is known as papilla. The minor calyces unitedto form major calyces. The major calyces, minor calyces, infundibula, and the renal pelvis are together called intrarenal collecting system [1]. Alterations in the gross anatomy of the renal collecting system are probably as numerous as that of fingerprints of individuals. The minor calyces and these major calyces drains into the infundibula of renal pelvis form each major calyx. This calyceal pattern forms the collecting system of the kidney [2]. Differences in the gross structure of the renal collecting system areas many as there are individuals and thus can be liked to fingerprints [3]. The bilateral collecting systems present in any single individual are rarely identical and may be quite different even from one another. The symmetry of the calyceal system in a single individual on both sides is only around 37% [4]. Not only the position and count of various parts of the collecting system differ between individuals, but also the parts can either be numerous or absent made a different type of classification of the pelvicalyceal patterns depending on the shape of the renal pelvis along with prominences of the calyces [5]. Renal collecting system injuries during percutaneous nephrolithotomy (PCN) occur in up to 8% patients [6]. A detailed knowledge of calyceal anatomy is very much essential for all endourological procedures, which is essential for the selection of the best suitable method for kidney stone removal and also this knowledge helps in better interpretation of standard intravenous urography [7]. Therefore, it is of interest to know the various patterns of major calyceal system and its frequency and its corresponding differences in number of minor calyces.

## Methods:

### Ethical clearance

The present study was done after getting approval from Institutional Human Ethics Committee, Saveetha Medical College and Hospital. The study was conducted in specialized scan center located in Chennai from individuals came for investigation for donating kidney after obtaining consent.

### Dataset

The renal diseases were ruled out in those individuals after blood investigations and ultrasound. This prospective cross-sectional study conducted on 99 fit kidney donors (male - 54, female - 45) using CT Angiogram. The donors were subjected to CT angiogram and the images were obtained. The CT machine used was light speed VCTXTe, ADW4.5 Version. 64 slice Computed Tomography Angiography. The digital CT angiographic images were taken and the renal pelvis with its branching pattern of the major and minor calyces and their corresponding cap of cortical tissues were analyzed. The major calyceal pattern was studied for Bi - Calyceal, Tri - Calyceal and Multi - Calyceal types.

### Statistical analysis:

The association of occurrence of calyceal pattern between male and female was compared analyzed using Chi square test. Comparison of number of minor calyces in three types of major calyceal pattern was analyzed using one-way ANOVA and between the sides student t test was used. P value of less than 0.005 was taken as significant.

## Results:

**Figure 2:**
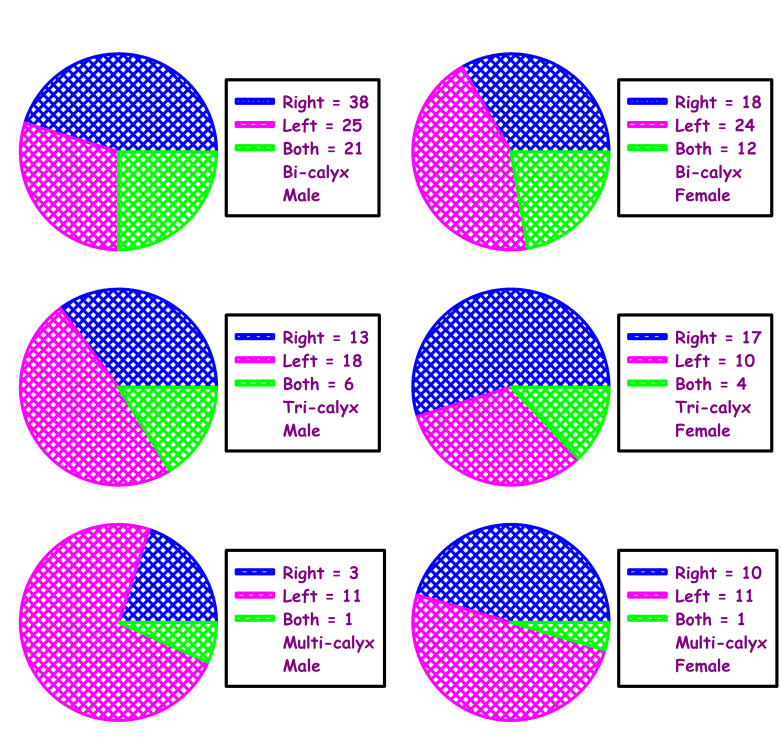
Association of occurrence of calyceal pattern of right, left and both sides of kidneys between male and female. Male = 54; Female = 45. Male (χ^2^) = 11.968; P < 0.018. Female (χ^2^) = 6.672; P < 0.154.

**Figure 3:**
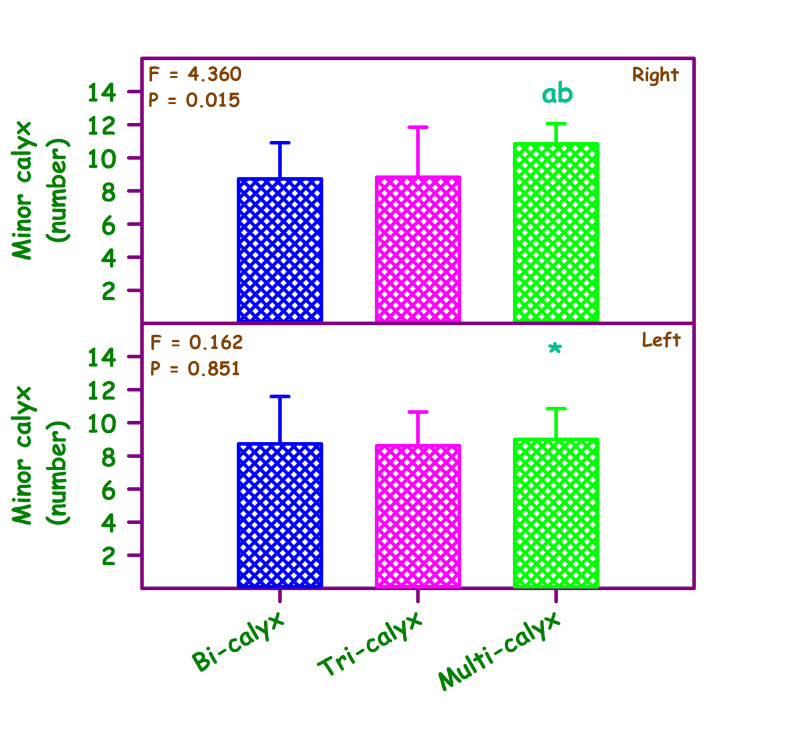
Comparison of number of minor calyx in different calyceal pattern. Right side F= 4.360, p<0.015; left side F = 0.162, p<0.851. Comparison was also made between right and left. ab significantly different from bicalyx and tricalyx – Right. *Significantly different multicalyx. Student t test was used to compare the sides. Bicalyx 't' Value = 0; 'p' Value = 1. Tricalyx 't' Value = 0.323; 'p' Value = 0.748. Multicalyx 't' Value = 3.207; 'p' Value = 0.003*.

In this study, pelvic-calyceal pattern in 198 kidneys (99 donors, both side) were analyzed for pelvi-calyceal pattern (Figure 1 - see PDF, Table 1 - see PDF). In this study all the renal pelvis was intra-renal and there was no extra-renal pelvis found. The study found 105 bi-calyceal pattern kidneys were found out of these 105 bi-calyceal kidneys, 56 were on right side, 49 were on left side. In this study 58 tri-calyceal pattern kidneys were found. In these 58, right side was 30, left side was 28. Multi-calyceal pattern was found in 35 kidneys; right side - 13; left side - 22. Also the association of occurrence of calyceal pattern between male and female were compared. There was significant association of occurrence in males (X^2^ = 11.968) with p value = 0.018 and there was no remarkable association in females (X^2^ = 6.672) (p = 0.154) (Figure 2, Table 1 - see PDF). This study also compared the total number of minor calyx in different major calyceal pattern on right side and left side by one-way ANOVA. There was significant difference in number of minor calyx in right side multi-calyceal type (Figure 3) than left side multi-calyceal type; the number of minor calyces was more on right side (p value=0.003). The bi and tri-calyceal type didn't show significant difference in number of minor calyx in right and left side.

## Discussion:

The significance of knowing pelvi-calyceal system will gain relevance with the advent of newer and effective treatment modalities and investigative procedure to diagnose pathologies involving kidneys. The literature available regarding pelvicalyceal pattern is less hence this study has done to provide anatomical data of various types of pelvi calyceal pattern in donors. Gandhi KR & Chavanhave studied the pelvicalyceal patterns in Brazilian population [4]. This study classified the pelvicalyceal system into two groups; Group A (Bi - calyceal pattern) and B. Group B has been further divided into two groups; Group B1 (Tri-calyceal pattern) and Group B2 (Multi-calyceal pattern). In the present study 53% of kidneys were of bi-calyceal pattern (Group A), 29% of kidneys were of tri-calyceal pattern (Group B1) and 18% of kidneys were multi-calyceal pattern (Group B2). Comparing these findings with another study done on human cadaveric kidney [8] they found 35% were bi-calyceal, 27% were tri-calyceal and 23% were multi-calyceal (Table 2 - see PDF). Another study done on intravenous urography showed multi-calyceal pattern was the most common type followed by bi-calyceal pattern whereas in the present study bi-calyceal pattern was the most common type. The present study provides an insight into the various patterns in live donors of South Indian population. This study also found the most common calyceal pattern among male and female kidneys. In males the right side bi-calyceal pattern is more common and in females left side bi-calyceal is more common. In tri-calyceal pattern, left side was more in males and right side was more common in females. In multi-calyceal pattern, left side percentage was more in both males and females. In addition to the pattern, this study also found the association of occurrence with this pattern in males and females. The association was significant in males and insignificant in females. In this study the quantity of minor calyces was compared with the major calyceal pattern. There was significant difference in minor calyces in right side multi-calyceal pattern than left side. In right side there was more number of minor calyces in multi-calyceal pattern and this was statistically significant. The remaining pattern (Bi and Tri-calyceal) doesn't show significant differences in number of minor calyces. More number of minor calyces can interrupt in endoscopic procedures complicating the surgeries leading to low success rate. A transcription factor, expressed in the metanephric blastema called the WT1 (Wilms Tumor Factor 1) and GDNF (Glial Derived Neurotropic Factor) stimulate the branching and growth of the ureteric buds. Also the ureteric bud divides early even before it comes in contact with the metanephric tissues [9]. The knowledge of the pelvi calyceal anatomy in relation to the variation in the number, arrangement of the minor and major calyces, position and presence of intra renal or extra renal pelvis is clinically important because huge number of developments have taken place in fields of endourology, percutaneous nephrolithotomy and various urologic procedures.[10] These procedures were done using cannula with the help of imaging techniques keeping in mind the different palvicalyceal pattern.

## Conclusion:

A depth anatomical knowledge of calyceal pattern is also important for donor selection. Regional anatomy is assessed in detail to decide the precise surgical method, which will avoid donor complication, and to ensure good recipient graft function. We show the different calyceal pattern among males and females and its distribution and association. The bi-calyceal pattern is the most common distribution and multicalyceal pattern is the least common pattern. The number of minor calyx is significantly more in right multi-calyceal type. A detailed description of calyceal pattern will be great significance in renal transplantation and also for other urological procedures.
